# What midwives should know about fertility awareness and its impact on reproductive behavior

**DOI:** 10.18332/ejm/195830

**Published:** 2025-01-03

**Authors:** Evangelia Saranti, Vicentia C. Harizopoulou, Eleni Bili, George Pados, Dimitrios G. Goulis, Dimitrios Vavilis, Victoria Vivilaki

**Affiliations:** 1 First Department of Obstetrics and Gynecology, Papageorgiou General Hospital, Medical School, Aristotle University of Thessaloniki, Thessaloniki, Greece; 2 Department of Midwifery, School of Health and Care Sciences, University of West Attica, Athens, Greece

**Keywords:** fertility, reproductive behavior, demographics

Fertility awareness methods inform women and couples about the menstrual cycle, ovulation, and fertility patterns with the aim to help individuals make informed decisions regarding family planning, conception, and contraception^1^. Thus, fertility awareness affects reproductive behavior. Although research has focused on women’s practices, knowledge, and attitudes regarding conception and fertility, non-medical variables, such as economic status, social factors, cultural values, and education level, needs to be studied more.

Midwives play a significant role in fertility awareness. According to essential competencies for basic midwifery practice, midwives support a holistic, respectful, and patient-centered approach to reproductive behavior. Moreover, they stress the importance of informed decision-making and work on empowering individuals and couples regarding their reproductive health^2^. Therefore, midwives should be familiar with fertility awareness and its impact on reproductive behavior and demographic indicators.

Fertility awareness can influence reproductive behavior by helping women trying to conceive to identify the fertile window. By tracking indicators, such as basal body temperature and cervical mucus, couples can time intercourse to coincide with ovulation, increasing the chances of conception. On the other hand, fertility awareness methods can be used as natural contraception. By identifying the fertile window, couples can choose to abstain from intercourse or use barrier methods as needed^1,3^. This knowledge offers women control over their fertility and the ability to make informed decisions about when to pursue or delay pregnancy. Couples can plan and space pregnancies according to their goals, achieve the desired family size, and avoid unintended pregnancies. Finally, fertility awareness promotes body literacy and enhances communication and shared responsibility within relationships^3,4^.

Reproductive behavior on the other hand plays a significant role in shaping demographics, as it directly influences population size, growth rate, age structure, and other demographic characteristics. Moreover, reproductive behavior can have long-term implications for a country’s demographic structure and socioeconomic dynamics^5,6^.

More specifically, high fertility rates (average number of children per woman) can lead to rapid population growth, a higher proportion of young people in the general population, and increased population density in certain regions. Conversely, low fertility rates can lead to population decline or slow growth, an aging population with an increased proportion of older individuals, and decreased population density, which creates the need for immigration to maintain the workforce. Finally, various demographic factors influence reproductive behavior, including cultural norms, access to contraceptives, education, economic conditions, and government policies.

## Contemporary reproductive behavior and demographic indicators

Nowadays, women are becoming mothers at an increasing older age, and there is a higher proportion of childless women in the 20–29 years age group (80%) than in the 30–39 years age group (34.4%)^7^. This trend is unfavorably affecting the age composition of the population. Developed countries, such as those in the European Union, are characterized by declining fertility rates. Greece, for instance, has one of the lowest fertility rates in Europe, with an average of 1.3 children per woman of childbearing age, while the European average is 2.1^7^. This decline significantly affects the population structure below the generational replacement threshold. Therefore, fertility awareness can improve reproductive behavior by providing information on knowledge, attitudes, and practices adopted by women^8,9^.

## Infertility and childbearing intention at older ages

Infertility is defined as the inability of a couple to achieve conception after 12 months of regular sexual intercourse without using contraceptives or the impairment of individuals’ ability to reproduce^10^. It affects between 12.6% and 17.8% of couples of reproductive age globally^11^, and one of the most well-established factors that can cause infertility is the advanced age of the woman^12,13^.

Nowadays, for many women, the age of the first child is postponed to an older age, and more women delay childbearing beyond the age of 30 years^14,15^. As a result, there is a reduced conception rate, an increase in fertility issues, and a greater need for medically assisted reproduction methods^14,16,17^. Nevertheless, the last factor cannot fully counteract the loss of fertility associated with older maternity age^18^.

The increasing childbearing age is related to social, economic, and personal factors, such as changing lifestyles, high educational demands, high cost of raising children, and increasing desire for a professional career^15,19^.

## Women’s knowledge and attitudes about fertility

Fertility knowledge is limited in the general population; as an example, the probability of conception during ovulation is overestimated^19^. Fertility awareness can be a means to improve the issues mentioned above, including the increased use of assisted reproduction methods and the formulation of realistic expectations during fertility treatments^20^. Moreover, fertility awareness can demonstrate the negative impact of specific lifestyles (e.g. smoking, excess alcohol, obesity) on fertility and provide ways to eliminate them^6,12^.

A recent study conducted in Canada highlighted women’s poor fertility knowledge. The study suggested that women must be educated about risk factors associated with conceiving at an older age and the potential problems related to conception, fertility, and perinatal risks for the mother and the infant^21^.

## Conclusion

The level of knowledge and the information sources about conception and fertility, are essential and crucial areas of investigation. There is a need for accurate information from reliable sources to educate the population on relevant issues systematically. In addition, healthcare professionals, and especially community midwives should discuss these issues at the optimal time of parenthood.

## Data Availability

Data sharing is not applicable to this article as no new data were created.
