# Erratum: What midwives should know about fertility awareness and its impact on reproductive behavior

**DOI:** 10.18332/ejm/200080

**Published:** 2025-01-17

**Authors:** European Publishing Production Team

**Affiliations:** 1 European Publishing Production Team, Heraklion, Greece

**Keywords:** fertility, reproductive behavior, demographics

**Erratum on: What midwives should know about fertility awareness and its impact on reproductive behavior**

**By Evangelia Saranti, Vicentia C. Harizopoulou, Eleni Bili, George Pados, Dimitrios G. Goulis, Dimitrios Vavilis, Victoria Vivilaki**

**European Journal of Midwifery, Volume 9, Issue January, Pages 1-3**

**Publish date: 3 January 2025**

**DOI:** https://doi.org/10.18332/ejm/195830

In the original article, the article number was mistakenly given as follows:

Eur J Midwifery 2025;9(January):77

The article number in the original manuscript is now corrected:

Eur J Midwifery 2025;9(January):1

The mentioned change is corrected also online.

